# Supplementary material for: Vascular Flora on Croatian Historic Structures: Drivers of Biodeterioration and Conservation Implications
Source: Plants (Basel). 2025 Jun 10;14(12):1773. doi: 10.3390/plants14121773 (PMC12197096; doi:10.3390/plants14121773)
Supplement: Supplementary file 1 [file plants-14-01773-s001.zip › Supplementary Table S1.pdf]

Table S1. Inventory of deteriogenic vascular flora at 40 study sites in Croatia.

Species	Family	Chorology	Plant Life Form	HI
Acer campestre L.	Sapindaceae	Europ.-Caucas.	P scap	10
Acer monspessulanum L.	Sapindaceae	Euri-Medit.	P scap	10
Adenostyles alpina (L.) Bluff & Fingerh.	Asteraceae	Orof. S-Europ	H scap	4
Adiantum capillus-veneris L.	Pteridaceae	Subtrop	G rhiz	3
Ailanthus altissima (Mill.) Swingle	Simaroubaceae	E-Asiat.	P scap	10
Ajuga chamaepitys (L.) Schreb.	Lamiaceae	Euri-Medit.	T scap	2
Alnus glutinosa (L.) Gaertn.	Betulaceae	Paleotemp.	P scap	9
Amaranthus deflexus L.	Amaranthaceae	S-Americ.	T scap	1
Antirrhinum majus L.	Plantaginaceae	W-Medit.	H scap	6
Arenaria serpyllifolia L.	Caryophyllaceae	Subcosmop.	T scap	1
Artemisia verlotiorum Lamotte	Asteraceae	E-Asiat.	H scap	4
Artemisia vulgaris L.	Asteraceae	Circumbor.	H scap	4
Asparagus acutifolius L.	Asparagaceae	Steno-Medit.	NP	6
Asplenium ceterach L.	Aspleniaceae	Eurasiat.	H ros	1
Asplenium onopteris L.	Aspleniaceae	Medit.-Macarones.	H ros	3
Asplenium ruta-muraria L.	Aspleniaceae	Circumbor.	H ros	1
Asplenium scolopendrium L.	Aspleniaceae	Circumbor.	H ros	3
Asplenium trichomanes L.	Aspleniaceae	Cosmop.	H ros	3
Aurinia sinuata (L.) Griseb.	Brassicaceae	Anfiadriat.	Ch suffr	4

Avena barbata Pott ex Link	Poaceae	Medit.-Turan.	T scap	2
Bromus madritensis L.	Poaceae	Euri-Medit.	T scap	1
Calystegia sepium (L.) R.Br.	Convolvulaceae	Paleotemp.	H scand	5
Campanula fenestrellata ssp. fenestrllata	Campanulaceae	Endem.-Illirico	H scap	3
Campanula fenestrellata ssp. istriaca (Feer) Damboldt	Campanulaceae	Endem.-Illirico	H scap	3
Campanula portenschlagiana Schult.	Campanulaceae	Illirica	H scap	3
Campanula pyramidalis L.	Campanulaceae	Illirica	H scap	3
Capparis spinosa L.	Capparaceae	Eurasiat.	NP	8
Carex halleriana Asso	Cyperaceae	Euri-Medit.	H caesp	2
Catapodium rigidum (L.) C.E.Hubb.	Poaceae	Euri-Medit.	T scap	1
Celtis australis L.	Cannabaceae	Euri-Medit.	P scap	10
Centaurea dalmatica A.Kern.	Asteraceae	Endem.-Dalmatica.	H scap	2
Centaurea spinosociliata Seenus	Asteraceae	Endem.-Dalmatica.	H bienn	2
Centaurium erythraea Rafn	Gentianaceae	Paleotemp.	H bienn	1
Chaenorhinum minus (L.) Lange	Plantaginaceae	Euri-Medit.	T scap	1
Chelidonium majus L.	Papaveraceae	Circumbor.	H scap	2
Cichorium intybus L.	Asteraceae	Cosmop.	H scap	2
Clematis flammula L.	Ranunculaceae	Euri-Medit.	P lian	7
Clematis vitalba L.	Ranunculaceae	Europ.-Caucas.	P lian	7
Clinopodium nepeta (L.) Kuntze	Lamiaceae	Medit.-Mont.	H scap	5
Convolvulus arvensis L.	Convolvulaceae	Cosmop.	G rhiz	2

Convolvulus cantabrica L.	Convolvulaceae	Euri-Medit.	H scap	2
Cornus sanguinea L.	Cornaceae	Eurasiat.	P caesp	5
Corylus avellana L.	Betulaceae	Europ.-Caucas.	P caesp	9
Cotinus coggygria Scop.	Anacardiaceae	S-Europ.	NP	8
Crepis sancta (L.) Bornm.	Asteraceae	Medit.-Turan.	T scap	2
Crithmum maritimum L.	Apiaceae	Euri-Medit.	Ch suffr	4
Cymbalaria muralis G.Gaertn., B.Mey. & Scherb.	Plantaginaceae	Subcosmop.	H caesp	4
Cynodon dactylon (L.) Pers.	Poaceae	Subcosmop.	H rept	4
Dactylis glomerata L.	Poaceae	Paleotemp.	H caesp	4
Digitaria sanguinalis (L.) Scop.	Poaceae	Cosmop.	T scap	1
Diplotaxis tenuifolia (L.) DC.	Brassicaceae	Submedit.	H scap	4
Dittrichia viscosa (L.) Greuter	Asteraceae	Euri-Medit.	H scap	5
Ecballium elaterium (L.) A.Rich.	Cucurbitaceae	Euri-Medit.	G bulb	2
Echium vulgare L.	Boraginaceae	Europ.	H bienn	2
Epilobium montanum L.	Onagraceae	Eurasiat.	H scap	4
Erigeron annuus (L.) Desf	Asteraceae	N-Americ.	T scap	2
Erigeron sumatrensis Retz.	Asteraceae	S-Americ.	T scap	2
Erysimum odoratum Ehrh.	Brassicaceae	Centroeurop.	H bienn	6
Eupatorium cannabinum L.	Asteraceae	Paleotemp.	H scap	4
Euphorbia amygdaloides L.	Euphorbiaceae	Europ.-Caucas.	Ch suffr	1
Euphorbia cyparissias L.	Euphorbiaceae	Europ.	H scap	2

Euphorbia maculata L.	Euphorbiaceae	N-Americ.	T rept	1
Euphorbia prostrata Aiton	Euphorbiaceae	N-Americ.	T rept	1
Fagus sylvatica L.	Fagaceae	Centroeurop.	P scap	10
Fallopia convolvulus (L.) Á.Löve	Polygonaceae	Cosmop.	T scand	3
Festuca myuros L.	Poaceae	Subcosmop.	T scap	1
Ficus carica L.	Moraceae	Medit.-Turan.	P scap	10
Fragaria vesca L.	Rosaceae	Cosmop.	H rept	2
Fraxinus ornus L.	Oleaceae	Euri-Medit.-Sett.	P caesp	9
Galium album Mill.	Rubiaceae	Eurasiat.	H scap	1
Galium aparine L.	Rubiaceae	Eurasiat.	T scap	1
Geranium molle L.	Geraniaceae	Subcosmop.	H scap	2
Hedera helix L.	Araliaceae	Submedit.	P lian	7
Helichrysum italicum (Roth) G.Don	Asteraceae	S-Europ.	Ch suffr	5
Heliotropium europaeum L.	Boraginaceae	Medit.-Turan.	T scap	1
Helminthotheca echioides (L.) Holub	Asteraceae	Euri-Medit.-Orient.	H bienn	2
Hippocrepis emerus (L.) Lassen	Fabaceae	Europ.-Caucas.	NP	8
Hyoscyamus albus L.	Solanaceae	Euri-Medit.	H bienn	2
Hypericum perforatum L.	Hypericaceae	Paleotrop.	H scap	4
Hypochaeris glabra L.	Asteraceae	Euri-Medit.	T scap	2
Lactuca muralis (L.) Gaertn.	Asteraceae	Europ.-Caucas.	H scap	2
Lepidium graminifolium L.	Brassicaceae	Euri-Medit.	H scap	1

Lolium rigidum Gaudin	Poaceae	Paleosubtrop.	T scap	3
Lotus hirsutus L.	Fabaceae	Euri-Medit.	Ch suffr	1
Lysimachia arvensis (L.) U.Manns & Anderb.	Primulaceae	Subcosmop.	T rept	1
Malva sylvestris L.	Malvaceae	Subcosmop.	H scap	5
Mercurialis annua L.	Euphorbiaceae	Paleotemp.	T scap	1
Micromeria juliana (L.) Benth. ex Rchb.	Lamiaceae	Steno-Medit.	Ch suffr	4
Nerium oleander L.	Apocynaceae	S-Medit.	P caesp	7
Oloptum miliaceum (L.) Röser & Hamasha	Poaceae	Medit.-Turan.	H caesp	4
Origanum vulgare L.	Lamiaceae	Eurasiat.	H scap	2
Ostrya carpinifolia Scop.	Betulaceae	Circumbor.	P scap	8
Oxalis corniculata L.	Oxalidaceae	Cosmop.	H rept	4
Paliurus spina-christi Mill.	Rhamnaceae	SE-Europ	P caesp	7
Papaver rhoeas L.	Papaveraceae	E-Medit.	T scap	1
Parietaria judaica L.	Urticaceae	W-Medit.-Macarones.	H scap	5
Parthenocissus quinquefolia (L.) Planch.	Vitaceae	N-Americ.	P lian	7
Pentanema squarrosum (L.) D.Gut.Larr., Santos-Vicente, Anderb., E.Rico & M.M.Mart.Ort.	Asteraceae	Centroeurop.	H bienn	3
Petrorhagia saxifraga (L.) Link	Caryophyllaceae	Euri-Medit.	H caesp	3
Picris hieracioides L.	Asteraceae	Eurosiber.	H scap	5
Pinus halepensis Mill.	Pinaceae	Steno-Medit.	P scap	10
Pistacia lentiscus L.	Anacardiaceae	Medit.-Macarones.	P caesp	8

Pistacia terebinthus L.	Anacardiaceae	Euri-Medit.	P caesp	8
Plantago coronopus L.	Plantaginaceae	Euri-Medit.	H ros	4
Plumbago europaea L.	Plumbaginaceae	Steno-Medit.	Ch suffr	5
Poa pratensis L.	Poaceae	Circumbor.	H caesp	1
Populus alba L.	Salicaceae	Paleotemp.	P scap	10
Populus nigra L.	Salicaceae	Paleotemp.	P scap	10
Portulaca oleracea L.	Portulacaceae	Subcosmop.	T scap	2
Primula vulgaris Huds.	Primulaceae	Europ.-Caucas.	H ros	4
Prunus mahaleb L.	Rosaceae	Europ.-Caucas.	P caesp	8
Pseudoturritis turrita (L.) Al-Shehbaz	Brassicaceae	S-Europ.	H bienn	4
Quercus ilex L.	Fagaceae	Steno-Medit.	P scap	9
Quercus pubescens Willd.	Fagaceae	Europ.	P scap	10
Reichardia picroides (L.) Roth	Asteraceae	Euri-Medit.	H scap	6
Rhamnus alpina L.	Rhamnaceae	Medit.-Mont.	P caesp	8
Robinia pseudoacacia L.	Fabaceae	N-Americ.	P scap	9
Rosa canina L.	Rosaceae	Paleotemp.	NP	7
Rubia peregrina L.	Rubiaceae	Medit.	P lian	6
Rubus caesius L.	Rosaceae	Eurasiat.	P caesp	8
Rubus ulmifolius Schott	Rosaceae	Euri-Medit.	P caesp	8
Sagina procumbens L.	Caryophyllaceae	Subcosmop.	H rept	2
Sambucus ebulus L.	Adoxaceae	Euri-Medit.	H scap	7

Sanguisorba minor Scop.	Rosaceae	Subcosmop.	H scap	3
Satureja montana L.	Lamiaceae	W-Medit.	Ch suffr	4
Scolymus hispanicus L.	Asteraceae	Euri-Medit.	H bienn	3
Scrophularia canina L.	Scrophulariaceae	Euri-Medit.	H scap	3
Sedum acre L.	Crassulaceae	Europ.-Caucas.	Ch succ	3
Sedum album L.	Crassulaceae	Euri-Medit.	Ch succ	4
Setaria pumila (Poir.) Roem. & Schult.	Poaceae	Subcosmop.	T scap	1
Setaria verticillata (L.) P.Beauv.	Poaceae	Cosmop.	T scap	1
Silene vulgaris (Moench) Garcke	Caryophyllaceae	Subcosmop.	H scap	3
Smilax aspera L.	Smilacaceae	Paleosubtrop.	P lian	7
Solanum dulcamara L.	Solanaceae	Paleotemp.	NP	3
Solanum nigrum L.	Solanaceae	Cosmop.	T scap	3
Sonchus asper (L.) Hill	Asteraceae	Subcosmop.	H bienn	5
Sonchus oleraceus L.	Asteraceae	Subcosmop.	T scap	4
Spartium junceum L.	Fabaceae	Euri-Medit.	P caesp	8
Symphytum tuberosum L.	Boraginaceae	SE-Europ.	G rhiz	5
Teucrium flavum L.	Lamiaceae	Steno-Medit.	Ch suffr	5
Teucrium polium L.	Lamiaceae	Steno-Medit.	Ch suffr	4
Tribulus terrestris L.	Zygophyllaceae	Cosmop.	T rept	2
Trifolium campestre Schreb.	Fabaceae	Paleotemp.	T scap	1
Trifolium repens L.	Fabaceae	Subcosmop.	H scap	5

Tussilago farfara L.	Asteraceae	Paleotemp.	G rhiz	4
Ulmus minor Mill.	Ulmaceae	Europ.-Caucas.	P scap	10
Umbilicus horizontalis (Guss.) DC.	Crassulaceae	Steno-Medit.	G bulb	3
Urtica dioica L.	Urticaceae	Subcosmop.	H scap	2
Verbascum phlomoides L.	Scrophulariaceae	Euri-Medit.	H bienn	6
Verbascum sinuatum L.	Scrophulariaceae	Euri-Medit.	H bienn	6
Verbena officinalis L.	Verbenaceae	Cosmop.	H scap	4
Veronica chamaedrys L.	Plantaginaceae	Eurosiber.	H scap	1
Veronica persica Poir.	Plantaginaceae	Subcosmop.	T scap	1
Viola reichenbachiana Jord. ex Boreau	Violaceae	Eurosiber.	H scap	4
