# Supplementary material for: Vascular Flora on Croatian Historic Structures: Drivers of Biodeterioration and Conservation Implications
Source: Plants (Basel). 2025 Jun 10;14(12):1773. doi: 10.3390/plants14121773 (PMC12197096; doi:10.3390/plants14121773)
Supplement: Supplementary file 1 [file plants-14-01773-s001.zip › Supplementary Table S2.pdf]

**Table S2**. Species distribution across 40 monuments, expressed as absolute frequencies. Values indicate the number of plots in which each species was recorded. Species are listed in decreasing order of total frequency across all sites. Site numbers (1–40) follow alphabetical order, with corresponding monument names listed as follows: (1) Amphitheatre, (2) Andautonia, (3) Benković Castle, (4) Bogliuno Castle, (5) Brinje Castle, (6) Budak Tower, (7) Chapel St. Nicolas, (8) Church Mirine, (9) Colmo Walls, (10) Drivenik Castle, (11) Duecastelli, (12) Fiume Castle, (13) Fort Bourguignon, (14) Fort Casoni Vecchi, (15) Fort Giorgio, (16) Fort Grosso, (17) Fort Munida, (18) Fort Punta Cristo, (19) Fort San Michele, (20) Fort Valmaggiore, (21) Garić Tower, (22) Gradec Castle, (23) Grobnik Castle, (24) Hreljin Castle, (25) Kličevica Castle, (26) Krk Castle, (27) Krk Walls, (28) Maškovića Residence, (29) Medvedgrad Castle, (30) Morosini Castle, (31) Nin Roman Temple, (32) Novigrad Castle, (33) Plomine Walls, (34) Pula Castle, (35) Salatić Castle, (36) Senj Walls, (37) Thermae Mirine, (38) Vrana Castle, (39) Vrbnik Tower, (40) Zelingrad Castle.

| **Species** | **1** | **2** | **3** | **4** | **5** | **6** | **7** | **8** | **9** | **10** | **11** | **12** | **13** | **14** | **15** | **16** | **17** | **18** | **19** | **20** | **21** | **22** | **23** | **24** | **25** | **26** | **27** | **28** | **29** | **30** | **31** | **32** | **33** | **34** | **35** | **36** | **37** | **38** | **39** | **40** | **TOT** |
|---|---|---|---|---|---|---|---|---|---|---|---|---|---|---|---|---|---|---|---|---|---|---|---|---|---|---|---|---|---|---|---|---|---|---|---|---|---|---|---|---|---|
| *Parietaria judaica* | 11 | | 11 | 6 | 11 | 8 | 7 | 11 | 8 | 6 | 9 | 9 | 8 | 9 | 7 | 6 | 3 | 4 | 7 | 4 | | 12 | 7 | 2 | 7 | 4 | 2 | 7 | | 8 | 7 | | 9 | 11 | 6 | 5 | | 9 | 5 | | 246 |
| *Hedera helix* | | | | 3 | | | 3 | 1 | 2 | 4 | 7 | 1 | 2 | 6 | 1 | | 6 | 2 | 5 | 5 | 1 | 4 | | | | | | | 1 | | | 3 | 2 | | 7 | 1 | | 1 | 1 | 8 | 77 |
| *Asplenium ceterach* | | | | 2 | | 2 | 11 | 5 | 4 | 1 | 1 | 2 | 1 | | 1 | | | | | | | 6 | 3 | 3 | 1 | | | 1 | | 1 | | | 2 | | | 5 | 1 | 2 | | | 55 |
| *Ficus carica* | | 1 | 1 | 1 | | | | 1 | 2 | 1 | 1 | | 1 | 3 | 1 | 3 | 5 | 3 | 8 | 2 | | | 1 | | 2 | | | 4 | | | | | 1 | | | | 1 | 1 | | | 44 |
| *Erigeron sumatrensis* | | | 2 | 5 | | | | | 1 | | 2 | | 1 | | 1 | | 1 | 3 | 1 | | | 2 | | | | | 1 | | | 1 | 3 | | 2 | | | 2 | 5 | 2 | | 3 | 38 |
| *Asplenium ruta-muraria* | | | 1 | | | | 2 | | | 3 | | | | | | 1 | | | | | 1 | | 2 | 4 | | | | | 7 | | | 4 | | | | 3 | 3 | 1 | | 2 | 34 |
| *Cymbalaria muralis* | 3 | | | 4 | | | | | 2 | 2 | 1 | | | | | 1 | | | | | | | 2 | 1 | | 3 | 3 | | | 6 | | | 1 | 2 | | | | | 1 | | 32 |
| *Petroragia saxifraga* | | | | 1 | 3 | | 2 | 3 | | 3 | 1 | 2 | 2 | | 2 | | | 1 | 1 | | | | | 1 | | | | | | | 1 | | 2 | | | 5 | | 1 | | | 31 |
| *Campanula pyramidalis* | | | | 7 | | | | | | 4 | | 3 | | | | | | | | | | | | 7 | | | 1 | | | | | | | | | 3 | | | 3 | | 28 |
| *Rubus ulmifolius* | | | | 1 | | | | 2 | | | 2 | | 1 | 2 | 3 | 2 | 1 | 3 | 1 | | 2 | | | | | | | | | | | | | | | | 3 | 3 | | | 26 |
| *Satureja montana* | | | | 4 | | | | | | 3 | 4 | | | | 4 | 6 | | 1 | 1 | | | | | 2 | | | | | | | | | | | | | | | | | 25 |
| *Dittrichia viscosa* | | | | | | | | | | | | | 2 | 2 | 4 | 5 | | 2 | 4 | | | | | | | | | | | | 1 | | | | | | | | | | 20 |
| *Sonchus asper* | 2 | 2 | | 1 | 2 | | | | | 1 | 1 | | 1 | | | | | | | | | 1 | | | 2 | | 1 | | | | | | 1 | | 2 | 1 | | | 1 | 1 | 20 |
| *Arenaria serpyllifolia* | | | | 3 | | | | 1 | | | | 1 | 2 | | | | | | | | 1 | | | | | | 1 | | | | 1 | | | | 1 | 3 | 3 | | | 1 | 18 |
| *Helichrysum italicum* | | | | 2 | | | | | | | 4 | | | | 5 | 2 | | 1 | 3 | | | | | | | | | | | | | | | | | | | 1 | | | 18 |
| *Reichardia picroides* | 1 | | 1 | | | | | | | | | | 1 | 2 | | 2 | | 2 | | | | 1 | | | | | 1 | | | | 4 | | | | | 2 | | | 1 | | 18 |
| *Micromeria juliana* | | | | | | 4 | | | | 2 | | | | | | | | | | | | | | | 9 | | | | | | | | | | | | | 2 | | | 17 |
| *Clinopodium nepeta* | | | | | | | | | | 2 | 1 | | | 1 | 1 | 1 | | 3 | 3 | | | | | | | | | | | | | | 2 | | | 2 | | | | | 16 |
| *Catapodium rigidum* | 1 | | | 1 | | | | 1 | | | 1 | 1 | | | 1 | | | | | | | | | | 1 | | 1 | | | | | | | 1 | | 2 | 3 | 1 | | | 15 |
| *Setaria verticillata* | | | | | | | | 1 | | 1 | | | 1 | | | | | | | | | 2 | 2 | | | | 1 | 1 | | | 2 | | | 2 | | | 2 | | | | 15 |
| *Verbascum sinuatum* | | | | | 5 | | | | | | | | | | 1 | | | | 2 | | | 1 | | | | | | | | | 2 | | | 1 | | | | 2 | | | 14 |
| *Bromus madritensis* | | | | | | | | 2 | | | 1 | | | | | | | | | | | 1 | | | 1 | | 1 | | | | 1 | | | | 2 | 1 | 1 | 1 | | | 12 |
| *Oxalis corniculata* | 1 | 1 | | | | | | 1 | | | | | | | | | | | | | | | | | | | | | | 1 | | | | | | | 8 | | | | 12 |
| *Campanula fenestrellata ssp. istriaca* | | | | 1 | | | | | | | | | | | | | | | | | | | | | | | | | | | | | 10 | | | | | | | | 11 |
| *Campanula portenschlagiana* | | | | | | | | | | 4 | | | | | | | | | | | | | | 1 | | | 1 | | | | | | | | | 5 | | | | | 11 |
| *Chelidonium majus* | | 1 | | | | | | | | | | | | | | | | | | | 2 | | | | | | | | 4 | | | 2 | | | | | | | | 2 | 11 |
| *Pinus halepensis* | | | | | | | | | | | | | | 2 | 2 | 2 | 1 | 1 | | 2 | | | | 1 | | | | | | | | | | | | | | | | | 11 |
| *Setaria pumila* | 1 | 5 | | | | | | | | | 2 | | | | | | | | 1 | | | | | | | | | | | | | | | | | | | | | 1 | 10 |
| *Clematis vitalba* | | 1 | | | | | | | 1 | | | | | | | | | | | | | 4 | | | | | 1 | | | | | 1 | | | | | | | 1 | | 9 |
| *Picris hieracioides* | | 2 | | 1 | | | | | | | 1 | | 1 | | | | | 1 | | | 1 | 1 | | | | | | | | | | | | | | | | | | 1 | 9 |
| *Quercus ilex* | | | | | | | | | | | | | 3 | | | 2 | 3 | | | 1 | | | | | | | | | | | | | | | | | | | | | 9 |
| *Sedum album* | | | | 3 | | | | | 1 | | | 1 | | | | | | | | | | | 3 | | | | | | | | | | | | | | | 1 | | | 9 |
| *Verbascum phlomoides* | | | | | | | | | | | | | | | | | | | | | | | | | | | 1 | | 2 | | | | | | | | 3 | | | 3 | 9 |

| | | | | | | | | | | | | | | | | | | | | | | | | | | | | | | | | | | | | | | | | | |
|---|---|---|---|---|---|---|---|---|---|---|---|---|---|---|---|---|---|---|---|---|---|---|---|---|---|---|---|---|---|---|---|---|---|---|---|---|---|---|---|---|---|
| *Euphorbia cyparissias* | | | | | | | | | | | 4 | | | | | | | | | | | | | | | | | | 4 | | | | | | | | | | | | 8 |
| *Geranium molle* | | | | | | 3 | 3 | | | | 1 | | | | | | | | | | | | | | | | | | | | | | | | | | | 1 | | | 8 |
| *Antirrhinum majus* | | | | | | | | | | | | 3 | | | | | | | | | | | 1 | | | 1 | 2 | | | | | | | | | | | | | | 7 |
| *Chaenorhinum minus* | | | | | | | | | | | | | | | | | | | | | | | 4 | | | | | | 1 | | | | | | | 2 | | | | | 7 |
| *Crepis sancta* | | | | | | | | | | | | | | | 1 | | | | 1 | | 2 | | | | | | | | 1 | | | 2 | | | | | | | | | 7 |
| *Dactylis glomerata* | | | | | | | | | | | | | | | | | | 3 | | | | 1 | | 2 | | | | | | | | | | | | | 1 | | | | 7 |
| *Festuca myuros* | | | | | | | 1 | 2 | | | | 1 | | | | | | | | | | | 1 | | | | | | | | | 1 | | | 1 | | | | | | 7 |
| *Fraxinus ornus* | | | | | | | | 1 | | | | | | | | | | | | | | 1 | | | | | | | | | | | | | | 1 | 1 | | | 3 | 7 |
| *Pistacia terebinthus* | | | | | | | | | | 1 | 1 | | | | | | | 1 | | | | | | 1 | 2 | | | | | | | | | | | | 1 | | | | 7 |
| *Sanguisorba minor* | | | | 1 | | | | | | | 1 | | 1 | | 1 | 1 | | | | | | | | | | | | | | | | | | | | 2 | | | | | 7 |
| *Campanula fenestrellata ssp. fenestrellata* | | | | | | | | | | | | | | | | | | | | | | | | | | | | | | | | | | | | | | | 6 | | 6 |
| *Digitaria sanguinalis* | 2 | | | | | | | | | | | | | | | | | | | | | | 1 | | | | | | | 1 | 1 | | | 1 | | | | | | | 6 |
| *Euphorbia prostrata* | 3 | | | | | | | | 1 | | | | | | | | | | | | | | | | | | | | | | | | | 2 | | | | | | | 6 |
| *Pistacia lentiscus* | 1 | | | | | | | | | | | | 1 | | 1 | | | 2 | | 1 | | | | | | | | | | | | | | | | | | | | | 6 |
| *Poa pratensis* | | | | | 3 | | | | | | | | | | | | | | | | 1 | | | | | | | | 1 | | | | | | | | | | | 1 | 6 |
| *Quercus pubescens* | | | | | | | 3 | | | | 1 | | | | | | | | | | | 1 | | | | | | | | | | | | | | | | | | 1 | 6 |
| *Robinia pseudoacacia* | | | | | | | | | | | | | | | | | | 1 | | | | 2 | | | | | | | | | | 3 | | | | | | | | | 6 |
| *Solanum nigrum* | 2 | | | | | | | | | | | 1 | | | | | | 1 | | | | | | | | | | | | | | | | 1 | | | 1 | | | | 6 |
| *Aurinia sinuata* | | | | | | 2 | | | | | | | | | | | | | | | | | | | | | | | | | | | | | | | | 3 | | | 5 |
| *Capparis spinosa* | | | | | | | | | | | | | | | | | | | | | | | | | | | | 2 | | | | | | | | | | 3 | | | 5 |
| *Celtis australis* | | | | | | | | | | | | | | 1 | | | | | 1 | | | | | | | | | | | | | | | | | | | 3 | | | 5 |
| *Crithmum maritimum* | | | | | | | | | | | | | | | | | | | | | | | | | | 4 | | | | | | | | | | | | | 1 | | 5 |
| *Diplotaxis tenuifolia* | | | | | | | | | | 1 | | | 1 | 1 | | | | | | | | | | | | | | | | | | | | 1 | | 1 | | | | | 5 |
| *Mercurialis annua* | 1 | | | | | | | | | | | | | | | | | | | | | | 1 | | | | | | | | | | | 2 | | | 1 | | | | 5 |
| *Sonchus oleraceus* | 2 | | | | | | | | | | | | | | | | | | | | | | | | | | | | | | | | | 3 | | | | | | | 5 |
| *Tribulus terrestris* | | | | | | | | | | | | | | | | | | | | | | | | | | | | 1 | | | 3 | | | | | 1 | | | | | 5 |
| *Adiantum capillus-veneris* | 2 | | | | | | | | | | | | 2 | | | | | | | | | | | | | | | | | | | | | | | | | | | | 4 |
| *Ailanthus altissima* | | | | | | | | | | | | | 1 | 1 | | | | | 2 | | | | | | | | | | | | | | | | | | | | | | 4 |
| *Arabis turrita* | | | | | | | | | | | | | | | | | | | | | | | | | | | | | | | | 4 | | | | | | | | | 4 |
| *Clematis flammula* | | | | | | | | | | | | | | | | | | | | | | | | | | | | | | | | | | | 4 | | | | | | 4 |
| *Cynodon dactylon* | | | 1 | | | | | | | | | | 1 | | | | | | | | | | | | | | | 1 | | | | | | | | | | 1 | | | 4 |
| *Epilobium montanum* | | | | | | | | | | | | | | | | | | | | | | | | | | | | | 2 | | | | | | | | | | | 2 | 4 |
| *Eupatorium cannabinum* | | | | | | | | | | | 1 | | | | | | | | | | | 1 | | | | | | | | | | 1 | | | | | | | | 1 | 4 |
| *Lepidium graminifolium* | | | | | | 3 | | | | | | | | | | | | | | | | | | | 1 | | | | | | | | | | | | | | | | 4 |
| *Paliurus spina-christi* | | | | | | | | | | | | | | | 1 | | | | | | | | | | 1 | | | | | | | | | | | | 1 | | | 1 | 4 |
| *Populus nigra* | | | | | | | | | | | | | | | | | | | | | | | | | | | | | | | | 1 | | | | | | | | 3 | 4 |
| *Rubia peregrina* | | | | | | | | | | | | | | | | 1 | 3 | | | | | | | | | | | | | | | | | | | | | | | | 4 |
| *Silene vulgaris* | | 2 | | | | | | | | | 1 | | | | | | | | | | | | | | | | | | | | | | | 1 | | | | | | | 4 |
| *Umbilicus horizontalis* | | | | | | | | | | | | | | | | | | | | | | | | | | | 3 | | | | | | | | | 1 | | | | | 4 |
| *Urtica dioica* | | | | | 1 | | | | | | | | | | | | | | | | | | | | | | | | 2 | | | 1 | | | | | | | | | 4 |
| *Convolvulus arvensis* | | 1 | | | | | | | | | | | | | | | | | | | | | | | | | | | | | | | | | | 1 | 1 | | | | 3 |

| | | | | | | | | | | | | | | | | | | | | | | | | | | | | | | | | | | | | | | | | | |
|---|---|---|---|---|---|---|---|---|---|---|---|---|---|---|---|---|---|---|---|---|---|---|---|---|---|---|---|---|---|---|---|---|---|---|---|---|---|---|---|---|---|
| *Corylus avellana* | | | | | | | | | | | | | | | | | | | | | | | | | | | | | | | | | | | | | | | | 3 | 3 |
| *Cotinus coggygria* | | | | | 1 | | | | | 1 | | | | | | | | | | | | | | 1 | | | | | | | | | | | | | | | | | 3 |
| *Fagus sylvatica* | | | | | | | | | | | | | | | | | | | | | | | | | | | | | | | | 2 | | | | | | | | 1 | 3 |
| *Fragaria vesca* | | | | | | | | | | | | | | | | | | | | | | | | | | | | | 1 | | | | | | | | | | | 2 | 3 |
| *Papaver rhoeas* | | | | | | | | | | | | | | | | | | | | | | | 1 | | | | 2 | | | | | | | | | | | | | | 3 |
| *Spartium junceum* | | | | | | | | | | | | | | | | 3 | | | | | | | | | | | | | | | | | | | | | | | | | 3 |
| *Teucrium flavum* | | | | | | | | | | | 1 | | | | | | | 2 | | | | | | | | | | | | | | | | | | | | | | | 3 |
| *Viola reichenbachiana* | | 2 | | | | | | | | | | | | | | | | | | | 1 | | | | | | | | | | | | | | | | | | | | 3 |
| *Acer campestre* | | | | | | | | | | | | | | | | | | | | | | | | | | | | | | | | | | 2 | | | | | | | 2 |
| *Artemisia verlotiorum* | | | | | | | | | | | | 2 | | | | | | | | | | | | | | | | | | | | | | | | | | | | | 2 |
| *Asparagus acutifolius* | | | | | | | | | | | | | | | | | | 1 | | 1 | | | | | | | | | | | | | | | | | | | | | 2 |
| *Calystegia sepium* | | | | | | | 1 | | | | | | | | | | | | | | | | | | | | | | | | | | | | | | | | 1 | | 2 |
| *Cornus sanguinea* | | | | | | | | | | | | | | | | | | | | | | | | | | | | | 1 | | | | | | | | | 1 | | | 2 |
| *Erysimum odoratum* | | | | | 2 | | | | | | | | | | | | | | | | | | | | | | | | | | | | | | | | | | | | 2 |
| *Galium aparine* | | | | | | | | | | | | | | | | | | | | | | | | | | | | | | | | | | | | | 1 | 1 | | | 2 |
| *Heliotropium europaeum* | | | | | | | | | | | | | | | | | | | | | | | | | | | | 2 | | | | | | | | | | | | | 2 |
| *Hyoscyamus albus* | | | | | | | | | | | | | | | | | | | | | | | | | | 2 | | | | | | | | | | | | | | | 2 |
| *Lolium rigidum* | | | | | | | | | | | | | | | | | | | | | 1 | | | | | | | | 1 | | | | | | | | | | | | 2 |
| *Lysimachia arvensis* | 1 | | | | | | | | | | | | | | | | | | | | | | | | | | | | | | 1 | | | | | | | | | | 2 |
| *Pentanema squarrosum* | | | | | | | | | | | | | | | | 1 | | | | | | | | | | | | | | | | 1 | | | | | | | | | 2 |
| *Populus alba* | | | | | | | | | | | | | | | | | | | | | | | | | | | | | 2 | | | | | | | | | | | | 2 |
| *Rosa canina* | | | | | 1 | | | | | | | | | | 1 | | | | | | | | | | | | | | | | | | | | | | | | | | 2 |
| *Rubus caesius* | | | | | | | | | | | | | | | | | | | | | | | | | | | | | 1 | | | 1 | | | | | | | | | 2 |
| *Scrophularia canina* | | | | | | | | | | 2 | | | | | | | | | | | | | | | | | | | | | | | | | | | | | | | 2 |
| *Smilax aspera* | | | | | | | | | | | | | | | 1 | | | | | | | | | | | | | | | | | | | | | | | 1 | | | 2 |
| *Teucrium polium* | | | | | | | | | | | | | | | | | | | | 1 | | | | | | | | | | | | | | | | | 1 | | | | 2 |
| *Trifolium repens* | | | | | | | | | | | | | | | | | | | | | | | | | | | | | | | | | | | | | | | | 2 | 2 |
| *Ulmus minor* | | | | | | | | 1 | | | | | | | | | | | | | | | | | | | | | | | | | 1 | | | | | | | | 2 |
| *Verbena officinalis* | | | | | | | | | | | | | | | | | | | | | 1 | | | | | | | | | | | | | 1 | | | | | | | 2 |
| *Acer monspessulanum* | | | | | | | | | | | | | | | | | | | | | | | | | | | | | | | | | | | | | 1 | | | | 1 |
| *Adenostyles alpina* | | | | | | | | | | | | | | | | | | | | | 1 | | | | | | | | | | | | | | | | | | | | 1 |
| *Ajuga chamaepitys* | | | | | | | | | | | | | | | | | | | | | | | | | | | | 1 | | | | | | | | | | | | | 1 |
| *Alnus glutinosa* | | | | | | | | | | | | | | | | | | | | | | | | | | | | | | | | 1 | | | | | | | | | 1 |
| *Amaranthus deflexus* | | | | | | | | | | | | | | | | | | | | | | | | | | | | | | | | | | | | 1 | | | | | 1 |
| *Artemisia vulgaris* | | | | | | | | | | | | | | | | | | | | | | | | | | | | | 1 | | | | | | | | | | | | 1 |
| *Asplenium onopteris* | | | | | | | | | | | | | | | | | | | | | | | | | | | | | | | | | | | | | | 1 | | | 1 |
| *Asplenium scolopendrium* | | | | | | | | | | | | | | | | | | | | | | | | | | | | | | | | 1 | | | | | | | | | 1 |
| *Avena barbata* | | | | | | | | | | | | | | | | | | | | | | | | 1 | | | | | | | | | | | | | | | | | 1 |
| *Carex halleriana* | | | | | | | | | | | 1 | | | | | | | | | | | | | | | | | | | | | | | | | | | | | | 1 |
| *Centaurea dalmatica* | | | | | | | | | | | | | | | | | | | | | | | | | | 1 | | | | | | | | | | | | | | | 1 |
| *Centaurea spinosociliata* | | | | | | | | | | | | | | | | | | | | | | | | | | | | | | | | | | | | 1 | | | | | 1 |

| | | | | | | | | | | | | | | | | | | | | | | | | | | | | | | | | | | | | | | | | | |
|---|---|---|---|---|---|---|---|---|---|---|---|---|---|---|---|---|---|---|---|---|---|---|---|---|---|---|---|---|---|---|---|---|---|---|---|---|---|---|---|---|---|
| *Centaurium erythraea* | | | | | | | | | | | | | | | | | | | 1 | | | | | | | | | | | | | | | | | | | | | | 1 |
| *Cichorium intibus* | | | | | | | | | | | 1 | | | | | | | | | | | | | | | | | | | | | | | | | | | | | | 1 |
| *Convolvulus cantabrica* | | | | | | | | | | | | | | | 1 | | | | | | | | | | | | | | | | | | | | | | | | | | 1 |
| *Ecballium elaterium* | | | | | | | | | | | | | | | | | | | | | | | | | | | | 1 | | | | | | | | | | | | | 1 |
| *Echium vulgare* | | | | | | | | | | | | | | | | | | | | | | | | | 1 | | | | | | | | | | | | | | | | 1 |
| *Euphorbia amygdaloides* | | | | | | | | 1 | | | | | | | | | | | | | | | | | | | | | | | | | | | | | | | | | 1 |
| *Euphorbia maculata* | | | | | | | | | | | | | | | | | | | | | | | | | | | | | | | | | | | | | 1 | | | | 1 |
| *Fallopia convolvulus* | | | | | | | | | | | | | | | | | | 1 | | | | | | | | | | | | | | | | | | | | | | | 1 |
| *Galium album* | | | | | | | | | | | | | | | | | | | | | | | | | | | | | 1 | | | | | | | | | | | | 1 |
| *Helminthotheca echioides* | | | | | | | | | | | | | | | | | | | | | | | | | | | | | | | 1 | | | | | | | | | | 1 |
| *Hippocrepis emerus* | | | | | | | | | | | | | | | | 1 | | | | | | | | | | | | | | | | | | | | | | | | | 1 |
| *Hypericum perforatum* | | | | | | | | | | | | 1 | | | | | | | | | | | | | | | | | | | | | | | | | | | | | 1 |
| *Hypochaeris glabra* | | | | | | | | | | | | | | | | | | | | | | | | | | | | | 1 | | | | | | | | | | | | 1 |
| *Lactuca muralis* | | | | | | | | | | | | | | | | | | | | | | | | | | | | | | | | 1 | | | | | | | | | 1 |
| *Lotus hirsutus* | | | | | | | | | | | | | | | | | | | | | | | | | | | | | | | | | | | | | | 1 | | | 1 |
| *Malva sylvestris* | | | | | | | | | | | | | | | | | | | | | | | | | | | | | | | | | | 1 | | | | | | | 1 |
| *Nerium oleander* | | | | | | | | | | | | 1 | | | | | | | | | | | | | | | | | | | | | | | | | | | | | 1 |
| *Oloptum miliaceum* | | | | | | | | | | | | | | | | | | | | | | | | | 1 | | | | | | | | | | | | | | | | 1 |
| *Origanum vulgare* | | | | | | | | | | | | | | | | | | | | | | | | | | | | | 1 | | | | | | | | | | | | 1 |
| *Ostrya carpinifolia* | | | | | | | | | | | | | | | | | | | | | | | | | | | | | | | | | | | | | | | | 1 | 1 |
| *Parthenocissus quinquefolia* | | | | | | | | | | | | | | | | | | | | | | | | | | | | | | | 1 | | | | | | | | | | 1 |
| *Plantago coronopus* | | | | | | | | | | | | | | | | | | | | | | | | | | | | | | | 1 | | | | | | | | | | 1 |
| *Plumbago europaea* | | | | | | | | | | | | | | | | | | | | | | | | | 1 | | | | | | | | | | | | | | | | 1 |
| *Portulaca oleracea* | | | | | | | | | | | | | | | | | | | | | | | | | | | | | | | 1 | | | | | | | | | | 1 |
| *Primula vulgaris* | | | | | | | | | | | 1 | | | | | | | | | | | | | | | | | | | | | | | | | | | | | | 1 |
| *Prunus mahaleb* | | | | | | | | | | | | | | | | | | | | | | | | 1 | | | | | | | | | | | | | | | | | 1 |
| *Rhamnus alpina* | | | | | | | | | | | | | | | | | | | | | | | 1 | | | | | | | | | | | | | | | | | | 1 |
| *Sagina procumbens* | | 1 | | | | | | | | | | | | | | | | | | | | | | | | | | | | | | | | | | | | | | | 1 |
| *Sambucus ebulus* | | | | | | | | | | | | | | | | | | | | | | | | | | | | | | | | | | | | | 1 | | | | 1 |
| *Scolymus hispanicus* | | | | | | | | | | | | | | | | | | | | | | | | | | | | | | | | | | | | | 1 | | | | 1 |
| *Sedum acre* | | | | | | | | | | | | | | | | | | | | | | | | | | | | | | | | | | | | 1 | | | | | 1 |
| *Solanum dulcamara* | | | | | | | | | | | | | | | | | | | | | | 1 | | | | | | | | | | | | | | | | | | | 1 |
| *Symphytum tuberosum* | | | | | | | | | | | | | | | | | | | | | | | | | | | | | | | | 1 | | | | | | | | | 1 |
| *Trifolium campestre* | | | | | | | | | | | | | | | | | | | | | | | | | 1 | | | | | | | | | | | | | | | | 1 |
| *Tussilago farfara* | | | | | | | | | | | | | | | | | | | | | | | | | | | | | | | | | | | | | | | | 1 | 1 |
| *Veronica chamaedrys* | | | | | | | | | | | | | | | | | | | | | | | | | | | | | | | | | | | | | | | | 1 | 1 |
| *Veronica persica* | | | | | | | | | | | | | | | | | | | | | | | | | | | | | | | 1 | | | | | | | | | | 1 |
| *Asplenium trichomanes* | | | | | | | | | | | | | | | | | | | | | | | | | | | | | | | | | | | | | | | | | 0 |
| *Erigeron annuus* | | | | | | | | | | | | | | | | | | | | | | | | | | | | | | | | | | | | | | | | | 0 |
